# An Arginine Deprivation Response Pathway Is Induced in *Leishmania* during Macrophage Invasion

**DOI:** 10.1371/journal.ppat.1005494

**Published:** 2016-04-04

**Authors:** Adele Goldman-Pinkovich, Caitlin Balno, Rona Strasser, Michal Zeituni-Molad, Keren Bendelak, Doris Rentsch, Moshe Ephros, Martin Wiese, Armando Jardim, Peter J. Myler, Dan Zilberstein

**Affiliations:** 1 Faculty of Biology, Technion—Israel Institute of Technology, Haifa, Haifa, Israel; 2 Institute of Parasitology, McGill University, Ste Anne de Bellevue, Quebec, Canada; 3 Carmel Medical Center and Faculty of Medicine, Technion,—Israel institute of Technology, Haifa, Israel; 4 The Smoler Proteomic Center, Faculty of Biology, Technion-Israel Institute of Technology, Haifa, Israel; 5 Institute of Plant Sciences, University of Bern, Bern, Switzerland; 6 Strathclyde Institute of Pharmacy and Biomedical Sciences, University of Strathclyde, Glasgow, United Kingdom; 7 Center for Infectious Disease Research, formerly Seattle Biomedical Research Institute, Seattle, Washington, United States of America; 8 Departments of Global Health and Biomedical Informatics & Medical Education, University of Washington, Seattle, Washington, United States of America; Imperial College London, UNITED KINGDOM

## Abstract

Amino acid sensing is an intracellular function that supports nutrient homeostasis, largely through controlled release of amino acids from lysosomal pools. The intracellular pathogen *Leishmania* resides and proliferates within human macrophage phagolysosomes. Here we describe a new pathway in *Leishmania* that specifically senses the extracellular levels of arginine, an amino acid that is essential for the parasite. During infection, the macrophage arginine pool is depleted due to its use to produce metabolites (NO and polyamines) that constitute part of the host defense response and its suppression, respectively. We found that parasites respond to this shortage of arginine by up-regulating expression and activity of the *Leishmania* arginine transporter (LdAAP3), as well as several other transporters. Our analysis indicates the parasite monitors arginine levels in the environment rather than the intracellular pools. Phosphoproteomics and genetic analysis indicates that the arginine-deprivation response is mediated through a mitogen-activated protein kinase-2-dependent signaling cascade.

## Author Summary

Protozoa of the genus *Leishmania* are the causative agents of leishmaniasis in humans. These parasites cycle between promastigotes in the sand fly mid-gut and amastigotes in phagolysosome of mammalian macrophages. During infection, host cells up-regulate nitric oxide while/or parasites induce expression of host arginase, both of which use arginine as a substrate. These elevated activities deplete macrophage arginine pools, a situation that invading *Leishmania* must overcome since it is an essential amino acid. *Leishmania donovani* imports exogenous arginine *via* a mono-specific amino acid transporter (AAP3) and utilizes it primarily through the polyamine pathway to provide precursors for trypanothione biosynthesis as well as hypusination of eukaryotic translation Initiation Factor 5A. Here we report the discovery of a pathway whereby *Leishmania* sense the lack of environmental arginine and respond with rapid up-regulation in the expression and activity of AAP3, as well as several other transporters. Significantly, this arginine deprivation response is also activated in parasites during macrophage infection. Phosphoproteomic analyses of *L*. *donovani* promastigotes have implicated a mitogen-activated protein kinase 2 (MPK2)-mediated signaling cascade in this response, and *L*. *mexicana* mutants lacking MPK2 are unable to respond to arginine deprivation. The arginine-sensing pathway might play an important role in *Leishmania* virulence and hence serve as target for drug development.

## Introduction

Protozoan parasites of the genus *Leishmania* are causative agents of a wide spectrum of human and veterinary diseases, with clinical manifestations ranging from lesions of the skin and mucous membranes to lethal visceral disease [1]. *Leishmania* species cause morbidity and mortality throughout large areas of the Old and New World, with ~350 million people in 88 countries at risk of visceral leishmaniasis. Approximately 500,000 new cases are diagnosed annually, ~10% of which result in death. Visceral leishmaniasis is highly endemic in parts of Sudan, India, Bangladesh, Nepal and Brazil [2]. Current anti-leishmanial drugs, which include pentavalent antimony, amphotericin B and miltefosine, are toxic and/or expensive [3].

*Leishmania* exhibit a digenetic life cycle including the extracellular promastigote and intracellular amastigote forms. Promastigote development occurs in the female sand fly alimentary tract, while differentiation into obligatory intracellular amastigotes occurs within the phagolysosome of mammalian macrophages [4, 5]. While promastigotes rely predominately on amino acid and sugar metabolism for survival, amastigotes rely mostly on β-oxidation of fatty acids and amino acid catabolism [6, 7]. Intracellular differentiation is mimicked in axenic culture by shifting promastigotes from an insect-like (26°C, pH 7.4) to a lysosome-like (37°C, pH 5.5 and 5% CO_2_) environment [7–9].

During *Leishmania* invasion, infected macrophages activate cytotoxic pathways in an attempt to kill the pathogen. These pathways include induction of nitric oxide (NO) biosynthesis, as well as release of radical oxygen species (ROS)[10]. To counteract these cytotoxic activities, *Leishmania* species have developed several intricate strategies as means of suppression of host responses. Lipophosphoglycan encapsulating metacyclic promastigotes (the infective form of the parasite typically found in the sand fly mid-gut or the stationary phase of *in vitro* cultures) inhibits H_2_O_2_ production during phagocytosis [11]. *Leishmania* infection up-regulates arginase I activity in host macrophages, thereby limiting arginine available for NO synthase. In addition, amastigotes increase production of anti-oxidants such as trypanothione, a diglutathione molecule bridged by the polyamine spermidine [12], and higher levels of ascorbate-dependent peroxidase guarantee reduction of the thiol group [13]. Trypanothione neutralizes ROS, enabling safe development of amastigotes inside macrophage phagolysosomes.

In *Leishmania*, arginine is the sole source for spermidine production and consequentially trypanothione biosynthesis [14]. Arginine, *via* spermidine, is also the source for hypusine synthesis, a unique precursor for the eukaryotic translation Initiation Factor 5A [15, 16]. Significant utilization of arginine by both macrophages and amastigotes represents a metabolic bottleneck critical in determining the outcome of *Leishmania* infection [17, 18]. *Leishmania* parasites acquire arginine *via* a high affinity, mono-specific transporter (amino acid permease 3, AAP3) responsible for translocating arginine into *Leishmania* cells [19, 20]. *Leishmania donovani* promastigotes respond to amino acid deprivation by activating arginine transport, evident by a four-fold increase of LdAAP3.2 mRNA (LinJ.31.0910) and protein levels [21]. Castilho-Martins et al. (2011) [20] showed that the homologue of LdAAP3 in *L*. *amazonensis* exhibits a similar response, likely due to external and internal sensing mechanisms. Arginine transport rate is also influenced by polyamine pathway activity, for which arginine is the sole precursor [21]. *L*. *donovani* mutants lacking either ornithine decarboxylase or spermidine synthase take up arginine at half the rate of wild type parasites, and their cellular pool of arginine is reduced similarly. This suggests that, at least in promastigotes, about half the accumulated arginine is directed to polyamine biosynthesis and arginine transport activity is regulated by this pathway.

Studies from recent years established that lysosomes act as the major cellular pool of amino acids in mammalian cells [22]. Sensors on lysosome membranes report amino acid sufficiency in this organelle by activating the mTORC1 pathway. We hypothesize that in addition to escaping immediate host immune response, *Leishmania* invade macrophage lysosomes to ensure safe growth inside the amino acid rich environment [23]. Hence, it is likely that transporters on the surface of *Leishmania* amastigotes influence amino acid content of phagolysosomes. Interestingly, kinetic analysis of the LdAAP3 protein indicated that its activity is optimal at pH 5.5 [19], suggesting arginine transport activity is optimal in amastigotes residing in phagolysosomes. We hypothesize that *Leishmania donovani* amastigotes express high levels of LdAAP3 and thereby compete for arginine within the host phagolysosome.

Here, we show that cultured *L*. *donovani* promastigotes and macrophage-inhabiting amastigotes sense depletion of arginine from the environment. Minutes after parasites detect lack of arginine, AAP3 mRNA and protein levels are significantly up-regulated, resulting in increased arginine transport. Genome-scale transcriptomics (RNA-seq) revealed consistent changes in gene expression associated with arginine deprivation, suggesting the presence of a coordinated arginine deprivation response (ADR). Interestingly, an *L*. *mexicana* mutant lacking MPK2 [24] did not respond to arginine deprivation, showing no indication of changes in AAP3 mRNA levels or arginine transport; while signs of deprivation response were restored in the add-back strain, indicating a normal ADR. Significantly, the ADR is also activated during macrophage infection; 48 hours after macrophage invasion, *LdAAP3* expression increases in an MPK2-dependent manner. Analyses upon macrophage invasion indicated activation and deactivation of additional genes affected by ADR in promastigotes. Together, our data indicates that upon arginine deprivation, an external sensor in *Leishmania* cells activates an MPK2-mediated signaling pathway that induces up-regulation of arginine transport. We postulate that ADR provides invading parasites with means to evaluate its new environment and rapidly adapt its metabolic capacity. The results support the notion that transport up-regulation by the arginine deprivation pathway plays a role in parasite development inside its host.

## Results

### Arginine deprivation response directs arginine to glycosomes via LdAAP3

**Fig 1:**
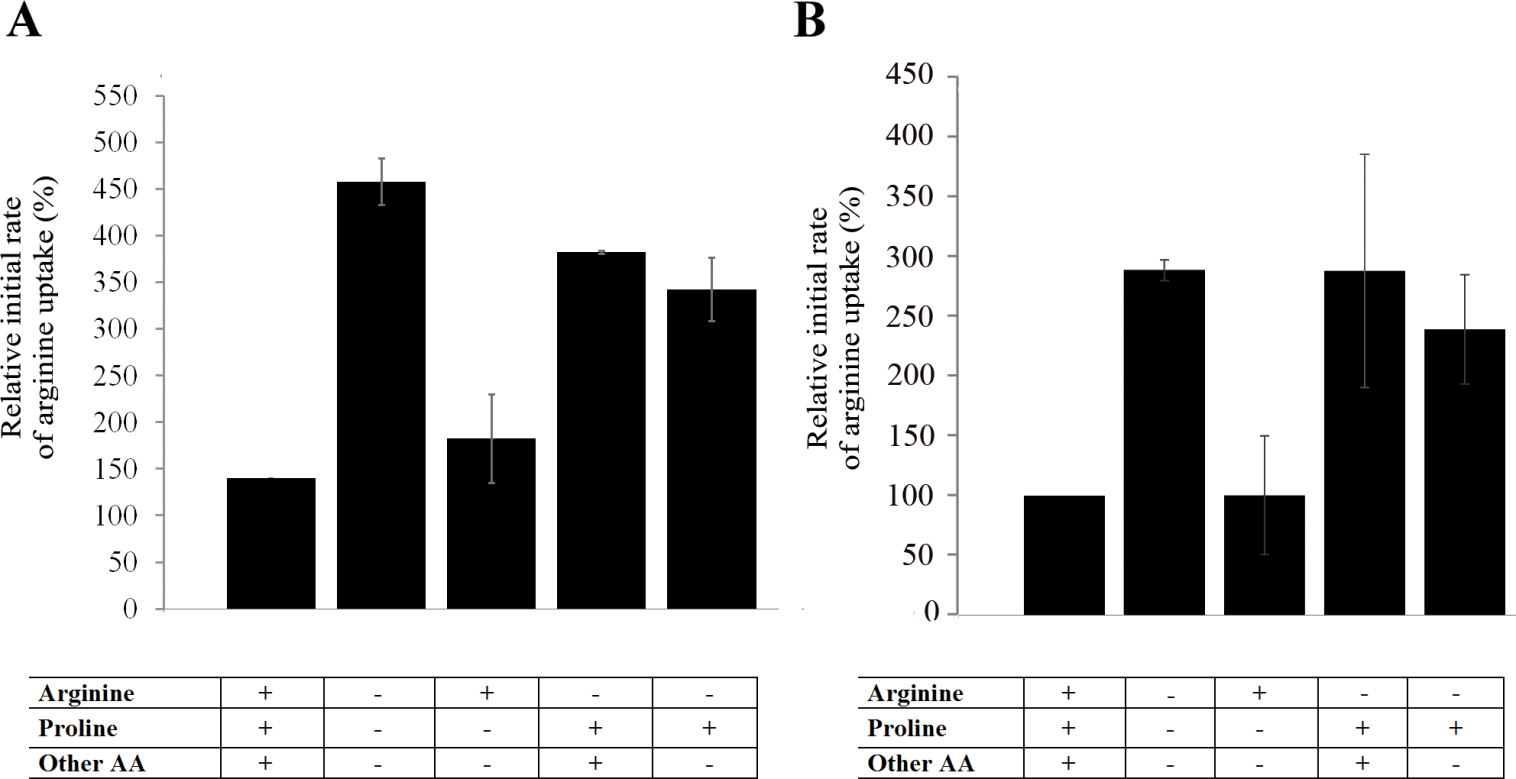
LdAAP3 response to deprivation is specific to arginine. Initial rates (up to 3 minutes) of arginine transport was determined in mid log-phase axenic *L*. *donovani* promastigotes (A) and amastigotes (B). Parasites (0.5-1X10^7^ cells/ml) were incubated for 2 hours in medium 199 (pH 7 and 5.5 for promastigotes and amastigotes, respectively) with or without the amino acids indicated in the X-axis of each panel. Subsequently, cells were washed twice in EARL's salt solution and subjected to arginine transport assays using the rapid filtration technique as described in Materials and Methods. Radiolabeled amino acids were added at medium 199 concentrations. Values are the mean±S.D. of three independent repeats. Transport rate at 100% was 0.4±0.05 nmol/min/1X10^8^ cells in promastigotes and 0.8±0.1 nmol/min/1X10^8^ cells in amastigotes.

Amino acid deprivation of *L*. *donovani* promastigotes results in up-regulation of arginine transport activity due to increased expression of *LdAAP3* [21]. We have now further explored the specificity of this response in both axenic promastigotes and amastigotes. As shown in Fig 1A, omission of only arginine from complete medium resulted in increased arginine transport by promastigotes, while addition of arginine to amino acid-free medium inhibited the response. Conversely, addition of proline only (or all other amino acids except arginine, not shown) had no effect on arginine transport rate. Similar results were observed in axenic amastigotes (Fig 1B). Thus, depletion of only arginine is necessary and sufficient for up-regulation of arginine transport in both promastigotes and amastigotes. The basal level of arginine transport was higher in amastigotes (0.8 nmol/10^8^ cells/min) than in promastigotes (0.4 nmol/10^8^ cells/min), further supporting the idea that this transport system is adapted to life in the acidic environment of phagolysosomes, as indicated previously.

**Fig 2:**
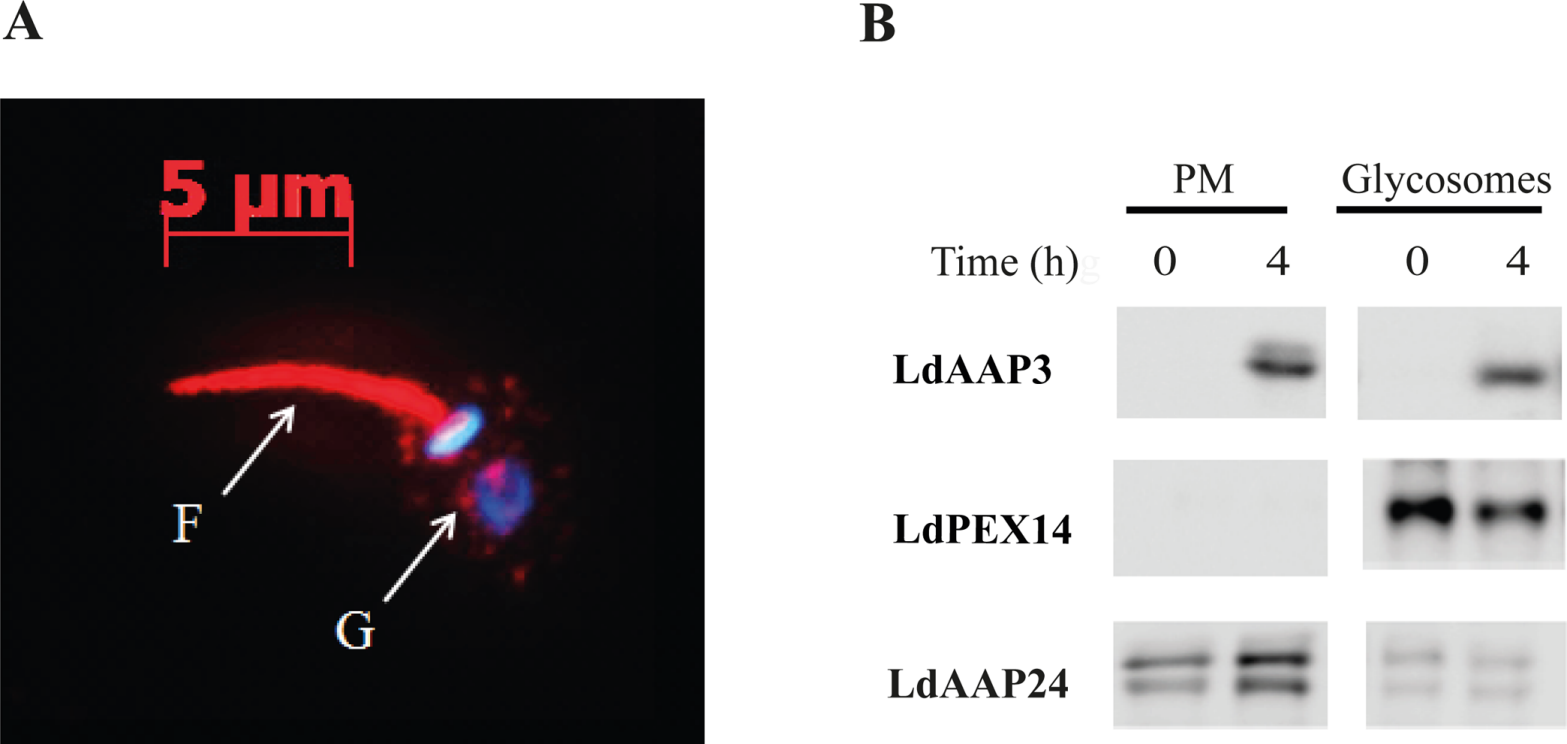
LdAAP3 localizes to both flagella surface and glycosome membranes. A) Indirect immunofluorescence of LdAAP3. Axenic *L*. *donovani* promastigotes were stained using anti LdAAP3 IgG (red) and the DNA stain DAPI (blue). The latter stained the nucleus and kinetoplast. The two immunofluorescence images were merged. Immunofluorescence analysis was performed using the inverted cell observer Zeiss Axiovert 2. B) Subcellular distribution of LdAAP3. Axenic *L*. *donovani* promastigotes before and after 4 hours of arginine deprivation were extracted, non-soluble membranes were separated and subsequently subjected to sucrose gradient (20–60%) as described in Materials and Methods. Aliquots of plasma and glycosome membrane fractions before and after arginine deprivation were subjected to western blot analysis. Anti proline/alanine transporter (LdAAP24) and PEX14 were used as plasma and glycosome membrane markers, respectively. LdAAP3 molecular mass is 46 kDa. Note that length of exposure to peroxidase chemiluminescense was calibrated to starved cells. Bands in non-starved cells were visible after longer exposure (not shown).

To supply the polyamine biosynthesis pathway, a portion of the arginine that is taken-up from the medium must be directed to the glycosomes, where arginase is localized [25, 26]. To assess whether, in addition to the plasma membrane, LdAAP3 also localizes to the glycosomal membrane, confocal immunofluorescence was carried out using anti-LdAAP3 antibodies. As shown in Fig 2A, LdAAP3 localized to the surface of the promastigote flagellum, as well as intracellular vesicles that are likely glycosomes. To verify this LdAAP3 localization, we performed cellular fractionation on a sucrose gradient, followed by protein separation on SDS-PAGE and western blot (Fig 2B). As shown, antibodies reacted with two cellular fractions; the plasma membrane (PM), identified using the *Leishmania* proline/alanine transporter LdAAP24 [27] and glycosomes, identified using LdPEX14 [28]. LdAAP3 abundance increased equally in both fractions following arginine deprivation, supporting the notion that arginine transport across the plasma membrane and into glycosomes is synchronized. Hence, the arginine deprivation response ensures that parasites produce polyamines even in environments with low arginine levels.

### LdAAP3 is rapidly up-regulated in response to low external arginine availability

**Fig 3:**
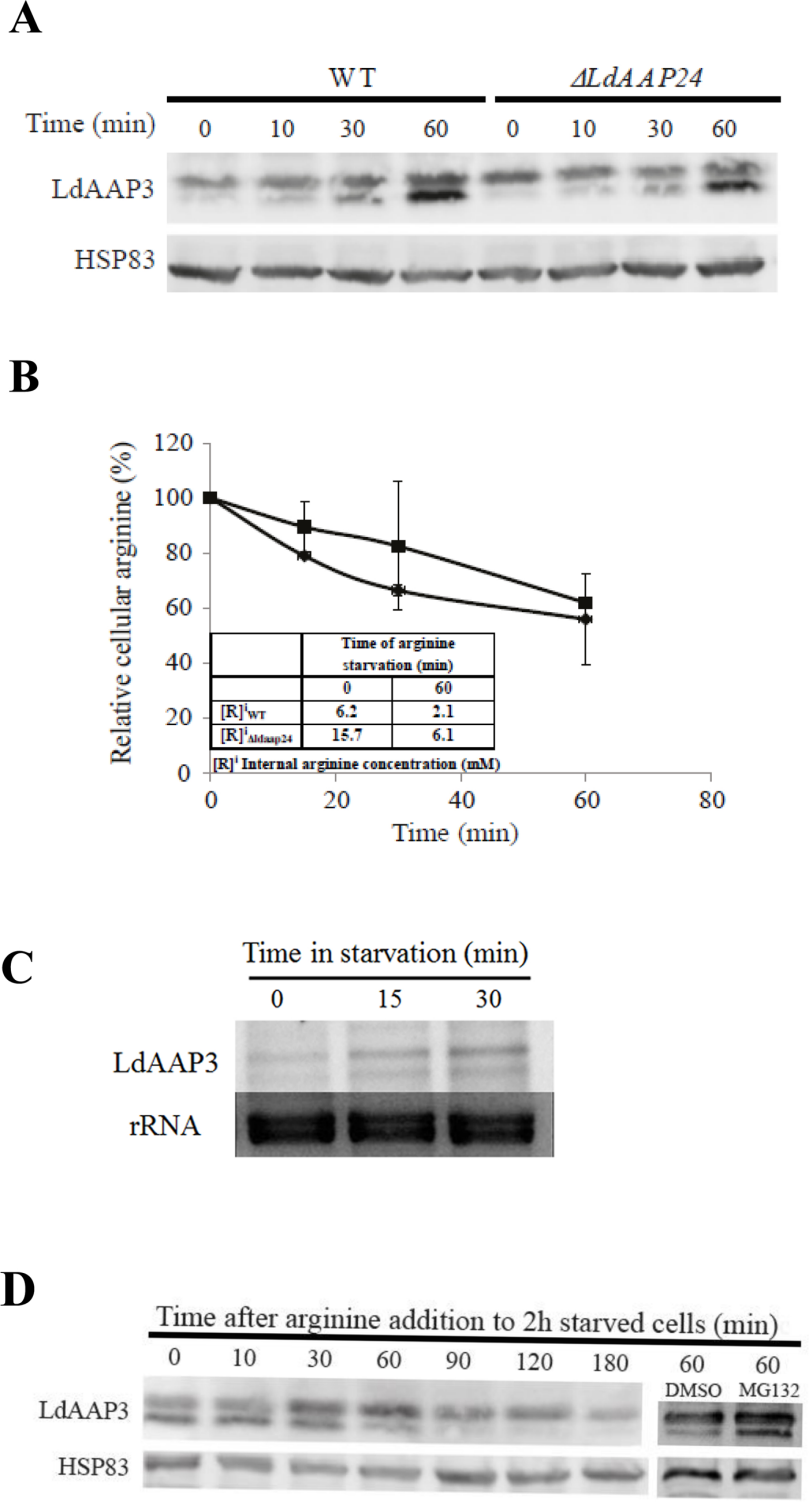
Time course of Arginine Deprivation Response (ADR). A) Time-course of LdAAP3 protein abundance increase as function of arginine deprivation time in wild type (left four lanes, lower bands) and *Δldaap24* (right four lanes, lower bands) *L*. *donovani* promastigotes. Mid-log phase parasites were suspended at a density of 0.5–1×10^7^ cells/ml in medium 199 without arginine and further incubated at 26°C. Aliquots were collected at 0, 10, 30 and 60 minutes, cellular proteins separated on 9% SDS-PAGE, transferred to nitrocellulose paper and subsequently probed with anti LdAAP3 antiserum. B) Rate of arginine pool depletion following arginine deprivation of *L*. *donovani* promastigotes wild type and *Δldaap24*. Aliquots of wild type (●) and *Δldaap24* (■) collected in (A) were subjected to amino acid analyses as described in Materials and Methods. Values are mean ± S.D. (n = 3). Inset is a table that indicates intracellular arginine concentration values at zero and 60 minutes. C) Northern analysis of *LdAAP3* mRNA abundance change during first 30 minutes of arginine deprivation. As a probe we used LdAAP3.2 (LinJ.31.0910). D) Arginine (0.45 mM) added to promastigotes after 2 hours of arginine deprivation induced rapid degradation of LdAAP3. Degradation was inhibited by the addition of 1 mM MG132 (Last two right lanes).

Time-course analysis of *L*. *donovani* response to arginine deprivation was conducted by exposing promastigotes to arginine-free medium and extracting proteins at different time points. As shown in Fig 3A (left 4 lanes, lower bands) increased LdAAP3 protein levels were detected within 10 minutes after deprivation was initiated. Northern blot analysis showed a similar time-course for increase in *LdAAP3*.*2* (*LinJ*.*31*.*0910*) mRNA (Fig 3C). In contrast, *LdAAP3*.*1* (*LinJ*.*31*.*0900*) mRNA levels were constant throughout the time course (data not shown).

**Fig 4:**
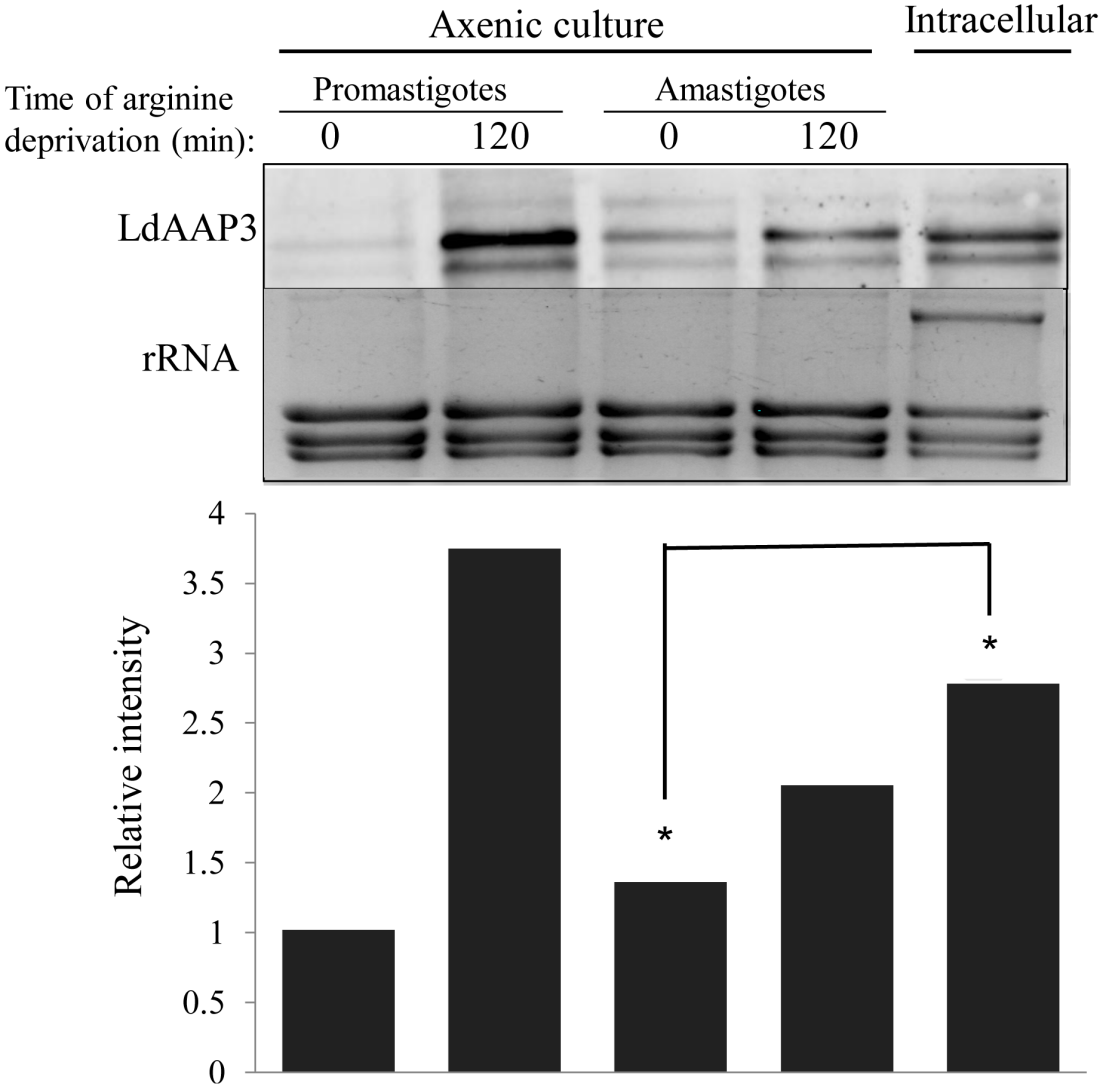
LdAAP3 expression increases during macrophage infection. LdAAP3.2 mRNA (LinJ.31.0910) abundance in axenic *L*. *donovani* promastigotes and amastigotes, before and after 2 hours arginine deprivation (indicated as "axenic culture") and THP1-derived amastigotes (indicated as "intracellular amastigotes"). Total RNA was extracted from axenic parasites and *L*. *donovani* infected-THP1 macrophages 48 hours after infection. THP1 macrophages were infected with late-log phase *L*. *donovani* promastigotes at a ratio of 10:1 parasites per macrophage. 48 hours after infection macrophages (30% infection, a mean of 3 parasites per macrophage) were collected and total RNA was extracted as described in Materials and Methods. *LdAAP3*.*2* was used as probe. Band abundance relative to mRNA of non-deprived promastigotes was calculated using TINA algorithm and are illustrated in the graphs below the gel.

This rapid response prompted us to identify the sidedness of arginine sensing. Recently, we found that the *L*. *donovani* mutant lacking the gene encoding alanine/proline transporter *LdAAP24* maintains a cellular arginine pool 2.6-times (15.5 mM) higher than wild type (6 mM) [27]. This suggests that if promastigotes are sensitive to change in intracellular arginine concentration, then the mutants’ response to arginine deprivation should be delayed compared to the wild type. We performed a time-course to monitor LdAAP3 response to arginine deprivation in *Δldaap24* using arginine-free medium (Figs 3A and 4 right lanes). As shown, LdAAP3 became visible 10 minutes after deprivation was initiated, identical to wild type. In parallel, we determined the change in the arginine pool as a function of time after exposure to arginine deprivation in wild type and mutant promastigotes (Fig 3B). We observed that arginine concentration in the mutant was higher than in wild type throughout the 60 minute assay, supporting the notion that arginine sensing is independent on the internal arginine concentration. These results together with our previous observation that LdAAP3 abundance is independent of the size of its arginine pool [21], indicate that *L*. *donovani* cells sense changes in external (*e*.*g*. growth medium) arginine. Interestingly, when arginine was added to medium two hours after deprivation was initiated, LdAAP3 abundance rapidly decreased (T_1/2_ of 60 minutes) to the endogenous level by a mechanism sensitive to the proteasome inhibitor MG132 (Fig 3D).

### Other transporters are also up-regulated by ADR

Because *LdAAP3*.*2* mRNA abundance paralleled up-regulation of LdAAP3 protein expression, we used genome-scale transcriptomic (RNA-seq) analysis to identify other mRNAs that showed similar up-regulation in response to arginine deprivation. RNA was extracted from promastigotes and axenic amastigotes before and after arginine deprivation for 15 and 120 minutes, respectively. PolyA^+^ mRNA was reverse-transcribed to prepare cDNA and sequenced using Illumina technology. Bioinformatic analysis of the resultant read-counts revealed that very few genes (29 and 9 in promastigotes and amastigotes, respectively) showed a more than 1.5-fold increase after arginine depletion. Moreover, only six mRNAs (including *LinJ*.*31*.*0910*, which encodes LdAAP3.2) increased by more than 1.3-fold in both promastigotes and amastigotes after arginine deprivation (Table 1). Interestingly, most of these encode membrane proteins; including a putative folate/biopterin transporter (LinJ.04.0020), a pteridine transporter (LinJ.10.1450), a sugar efflux transporter (LinJ.28.0490) and a major facilitator superfamily transporter (LinJ.36.2900). The only up-regulated mRNA that encodes non-membrane protein is a CCCH Zn finger protein, which is a putative RNA binding protein (LinJ.04.0040). We propose these genes are part of an arginine deprivation response (ADR) active in both promastigotes and amastigotes.

**Table 1:** Potential members of the ADR pathway.

GeneID	Putative function	Pro_15_	Ax_120_	Am
LinJ.04.0020	folate/biopterin transporter	1.5***	1.8***	1.2
LinJ.04.0040	RNA binding protein	1.4	1.8***	1.8***
LinJ.10.1450	pteridine transporter, putative	2.5***	3.8***	1.4*
LinJ.22.1550	membrane protein	1.5	1.4	2.4*
LinJ.28.0490	sugar efflux transporter	1.4	2.0**	1.3
LinJ.31.0910	amino acid permease 3 (AAP3)	1.3	2.0***	1.5***
LinJ.36.2900	MFS family transporter	2.4***	4.7***	1.8***

***p<0.001

**p<0.01

*p<0.05

Total RNA was isolated from two biological replicates of promastigotes (Pro) and axenic amastigotes (Ax) grown in the absence of arginine for 15 and 120 min, respectively; and intracellular amastigotes (Am) obtained 48h after infection of THP-1 macrophages with late-log promastigotes. RNA-seq libraries were sequenced on an Illumina Hi-Seq 2000 to obtain ~15 million 35-nt single-end reads from each library, aligned against the *L*. *infantum* JCPM5 genome and the reads counts for each CDS normalized using EdgeR. Fold-changes (relative to promastigotes grown in complete medium) are shown for all genes with >1.3-fold up-regulation in the absence of arginine in both promastigotes and axenic amastigotes; p-values are indicated by asterisks. The value for AAP3 is likely an under-estimate, since it represents reads from both *LinJ*.*31*.*0900* (*AAP3*.*1*) and *LinJ*.*31*.*0910* (*AAP3*.*2*). Manual inspection indicated >2-fold increase (under all conditions) of reads that map uniquely to the 5’ UTR of *AAP3*.*2*.

### Arginine deprivation response is induced during macrophage infection

Previously, we hypothesized that during infection, the parasite employs LdAAP3 to compete with its host macrophage for arginine by depleting the amino acid from phagolysosomes [21]. This implies that upon infection, the parasite depletes arginine in the phagolysosome of the host macrophage; activating the arginine deprivation response in the infecting amastigotes, leading to subsequent up-regulation of LdAAP3.2 (and the ADR proteins identified above). To assess this, we used RNA-seq analysis of intracellular amastigotes 48 hours after infection of THP-1 macrophages. As expected from our previous experiments [29, 30], 881 mRNAs showed more than 1.5-fold increase in abundance compared to promastigotes, while 888 decreased. Significantly, all six of the ADR-associated mRNAs described above increased in abundance in intracellular amastigotes (Table 1), although the changes were generally somewhat less than that seen for arginine depletion in the axenic system. Northern analysis validated these results by showing that *LdAAP3*.*2* mRNA in intracellular amastigotes was ~2–3 times more abundant than in non-deprived axenic amastigotes or promastigotes (Fig 4). Thus, it appears that the ADR is activated in intracellular amastigotes within 48 hours of macrophage infection.

### Arginine deprivation induces a Mitogen-Activated Protein Kinase 2-mediated pathway

As indicated above, *Leishmania* cells sense changes in the external arginine concentration. To induce an intracellular response, the sensor likely activates a signaling pathway that results in increased LdAAP3 expression and activity. To elucidate phosphoproteins involved in this pathway, we used the di-methylation tagging technique to examine changes in the phosphorylation profile of promastigotes after 5 and 15 minutes of arginine deprivation. These analyses identified 150 (in 110 proteins) and 122 (in 97 proteins) sites whose phosphorylation changed following arginine deprivation at the 5 and 15 minute time-points, respectively (S1 and S2 Tables). Interestingly, the vast majority of these phosphorylation sites changed in abundance at 5 (119/150) and/or 15 (97/122) minutes. More sites (71) increased in abundance at 5 minutes than decreased (48); while the reciprocal (40 up and 57 down) occurred at 15 minutes (the entire phosphorylation Data are available via ProteomeXchange with identifier PXD002830).

Remarkably, we found the most abundant phosphorylation motif was “SP”, appearing in 37% of the phosphopeptides at 5 minutes and 36% at 15 minutes. Recent phosphorylation analysis showed that 14.5% of phosphosites in non-deprived promastigotes were on an “SP” motif [31, 32], indicating the level of phosphorylation on this motif is significantly higher in arginine deprived cells. The SP motif is a major substrate of mitogen-activated protein kinases [33]. Analysis at 5 minutes arginine deprivation revealed five protein kinases whose phosphorylation abundance increased by >2-fold (Table 2 and S2 Table). One of these was a MPK2 homolog (LinJ.36.0780) that was phosphorylated at four sites in two peptides, two serines in the first, as well as threonine and tyrosine on the well-known “TxY” motif [34] in the second. The analysis also revealed that phosphorylation of a MPK10 homolog was reduced by >2-fold at 5 minutes. Since MPKs are usually activated by phosphorylation, we decided to focus on *Leishmania* MPK2 and assess its role in the ADR.

**Table 2:** Arginine deprivation-induced protein phosphorylation in *L*. *donovani* promastigotes.

Accession	Description	Phosphorylated tryptic peptide	Repeat 1	Repeat 2
			LOG2 fold from non-starved	
LinJ.25.2450*	AGC family serine/threonine protein kinase, putative	LFGGF**p**SCTADSHLNNS	2	2.99
LinJ.32.0270*	serine/threonine-protein kinase Nek1, putative	RGAPPGEPA**p**SRP**p**TTPQQQR	0.8	0.75
LinJ.34.2680*	PKA regulatory subunit of protein kinase a-R'	TIQMQRP**p**SR	1.19	1.06
LinJ.36.0780*	MPK2 mitogen-activated protein kinase	KT**p**SVS**p**SASAGGSR	1.1	0.99
LinJ.36.0780*	MPK2 mitogen-activated protein kinase	SILSLEGEQASRPVL**p**TD**p**YIATR	0.69	0.78
LinJ.15.0810**	protein kinase, putative	APPVSYNEPGHEQLD**p**SR	6.55	1.73
LinJ.27.1700**	protein kinase-like protein	G**p**SPDVDDKFVDVGDATK	6.67	1.68
LinJ.35.4060**	protein kinase A catalytic subunit isoform 1	SPGDTSNFEKYPD**p**SPVDR	1.19	1.13

* Protein kinase phosphorylation 5 minutes after arginine deprivation initiated

** Protein kinase phosphorylation 15 minutes after arginine deprivation initiated.

Di methylation of tryptic peptides extracted from *L*. *donovani* promastigotes 5 and 15 min after arginine deprivation initiated. Phosphopeptide enrichment, mass spectrometry and data analysis is described in detail in Materials and Methods.

**Fig 5:**
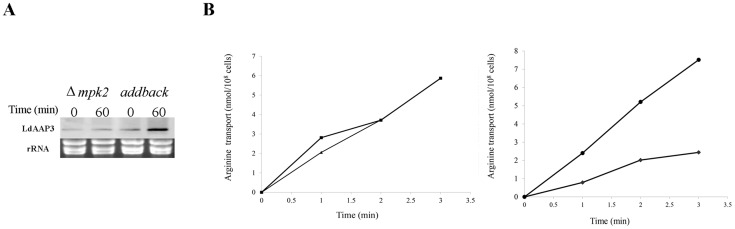
Arginine deprivation response is mediated by MPK2. Promastigotes of *L*. *mexicana* mutants lacking both copies of the MPK2 gene *(Δmpk2*) and those containing a single wild type copy of the gene re-inserted into their genome (addback) were subjected to 2 hours arginine deprivation as described in Fig 1. A) Northern analysis of total mRNA from these cells using *LmxMPK2* probe. B) Arginine transport was measured for *Δlmxmpk2* (left panel) and addback (right panel) cells grown in the presence (♦) or absence (•) of arginine.

Recently, Mandal et al. [24] deleted the gene encoding MPK2 from the *L*. *mexicana* genome and showed it mediates activation of aquaglyceroporin 1. Since the ADR phenomenon is universal among Old and New World *Leishmania* species [20, 21], we used this *L*. *mexicana* mutant to assess MPK2 mediation of ADR. *Δlmxmpk2* mutant promastigotes and a cell line with the wild type gene inserted back in the parasite genome (add-back) were exposed to 4 hours of arginine deprivation. Northern analysis (Fig 5A) showed that *Δlmxmpk2* cells did not up-regulate *LmxAAP3*.*2* expression and thus lacked the ADR, while add-back cells (expressing the wild type *MPK2* gene) elicited a pronounced response (>2-fold increase in *LmxAAP3*.*2* mRNA) to arginine deprivation. Furthermore, the initial rate of arginine transport in the *Δlmxmpk2* mutant was similar before and after deprivation, while add-back promastigotes responded significantly to amino acid deprivation (Fig 5B). Interestingly, the rate of arginine transport in the *Δlmxmpk2* mutant is only slightly lower than that of arginine-deprived (and higher than that of arginine-replete) add-back and wild type cells (data not shown). These results further support an important role for MPK2 in the regulation of LmxAAP3 activity and the ADR in *Leishmania*.

## Discussion

In this study, we established that *Leishmania* cells sense the absence of arginine in their environment; both in culture (axenic promastigotes and amastigotes) and in macrophages during infection (amastigotes). The sensor activates a MPK2-mediated response pathway (Arginine Deprivation Response) that induces arginine transport up-regulation *via* an mRNA stability mechanism. ADR induces co-expression of an additional six genes, five of which also encode membrane transporters. Thus, an external arginine sensor provides the invading parasite with the tools necessary to evaluate its environment and the means for subsequent activation of rapid adaptation to the metabolic challenge it faces in its new home within the phagolysosome.

Amino acid transporters with dual roles in sensing and transporting (transceptors) that monitor extracellular nutrient availability have been identified previously in yeast [35] and mammalian cells [36]. The most characterized is the amino acid sensor SSY1, a transceptor on the surface of *Saccharomyces cerevisiae* [35, 37–39]. Extracellular amino acids bind SSY1, leading to activation of the mTOR signal transduction pathway, activating transcription factors STP1 and STP2, which subsequently activate expression of several amino acid transporter genes [40, 41]. SNAT2 is a mammalian transceptor that transports neutral amino acid and senses amino acid availability [36]. The long soluble N-terminus of SNAT2 senses intracellular amino acid content thereby stabilizes the amino acid pool, while the short extracellular C-terminus regulates transport activity [42]. In contrast to the yeast and mammalian transceptors, which have low specificity and respond to amino acid availability, the *Leishmania* arginine sensor responds only to arginine availability. Whether LdAAP3, which has high amino acid specificity, is an arginine transceptor remains a mystery that we hope to solve in the near future.

A common feature of amino acid sufficiency sensors is that they transmit their signals by activating mTOR signaling pathways [43]. Specifically, in mammalian cells mTOR complex 1 (mTORC1) is activated in response to increased lysosome amino acid availability [22]. In contrast, arginine sensing in *Leishmania* (as described in this paper) reports on amino acid deprivation. This is likely the reason ADR is mediated by a distinct pathway namely MPK2 pathway, not mTOR.

Lysosomes have been recently recognized as a major location of the amino acid pool in mammalian cells, with sensors on the surface that report on lysosome amino acid sufficiency. Specifically, SLC38A9, a low affinity arginine/glutamine transceptor that localizes to lysosome membrane is an arginine sensor that activates the mTORC1 pathway when arginine concentration is high [44–46]. Interestingly, the mTOR pathway plays an important role in macrophage polarization during infection, regulating type 2 immunity, inflammation and allergy. When the mTORC1 pathway is activated, it induces the proinflammatory m1 response (as do INFγ and LPS) that induces production of cytotoxic NO and ROS, both of which fight parasite invasion. The *Leishmania* surface protease gp63 plays a role in preventing this activation by cleaving mTOR (Jaramillo et al, 2011). In addition, *Leishmania* infection induces an IL10/IL12 axis that inhibits mTORC1 activation, enabling an m2 response that (together with IL4, IL13 and TGFβ) stimulates macrophage arginase 1 to convert cytosolic arginine to ornithine and thereby limit NO synthesis [47].

In light of the above, we hypothesize that following invasion, *Leishmania* parasites actively compete for the diminishing arginine pool in the phagolysosome. When arginine concentration becomes low enough to prevent mTORC1 activation via the SLC38A9 pathway, the ADR is induced to up-regulate LdAAP3 expression, enabling parasite survival in this low arginine environment.

An interesting unexpected observation made in this study is the dual localizations of LdAAP3. Our analysis indicated that the protein in both localizations have identical N- and C-termini, neither of which contain a canonical glycosome localization motif. A similar phenomenon was observed for *Trypanosoma brucei* hexokinase [48], where proteins had the PTS2 signal peptide at their N-terminus, but localized to both the glycosome and cytosol. The predominant localization of LdAAP3 to the flagellar surface is not unique. Piper et al. [49] showed that the *Leishmania* Glut2 glucose transporter is primarily localized to the surface of the flagellum. Interestingly, both Glut2 and LdAAP3 are also expressed (albeit at low levels) on cell surface [19]. The fact that LdAAP3 is localized to both surface and glycosomal membranes strongly suggest that most of the arginine that is taken up from outside is directed to the glycosome, where arginase, the first enzyme in the polyamine pathway, resides.

This study describes the first amino acid deprivation sensing mechanism and the pathway that transduces this response, and reveals a novel host-pathogen metabolic interplay, which warrants further investigation.

## Materials and Methods

### Axenic *Leishmania* cell cultures

*L*. *donovani*, MHOM/SD/00/1S cell line was used [8]. Promastigotes and amastigotes were grown and maintained in axenic cultures. Differentiation was performed as previously described [50]. Briefly, late log promastigotes were transferred from promastigote medium at 26°C and pH 7.4 to amastigote medium at 37°C, pH 5.5 in 5% CO2 environment. Twenty-four hours after initiation of differentiation, cells were diluted 1:10 in pre-warmed amastigote medium.

### *In-vivo* infection of macrophages

THP1 macrophages were grown on a plate in 37°C in 5% CO_2_ incubator at a density of 10^6^ cells/plate. Late log-phase *L*. *donovani* promastigotes were added to the culture for 4 hours at 1:10 macrophage to parasite ratio. Plates were then washed three times with phosphate buffered saline (PBS) and incubation continued for 48 hours. Subsequently, infected macrophages were collected with a scraper, washed with PBS and centrifuged for 4 minutes at 48Xg. After Giemsa staining (Sigma Inc. USA) the nuclei of both macrophages and parasites takes on the characteristic magenta coloration, while the cytosol was colored blue. Infected macrophages and infection ratio per macrophage were calculated.

### Purification of macrophage-derived amastigotes

THP1 macrophages were grown on 8 plates at 37°C at a density of 1×10^7^ cells/plate. Mid log-phase *L*. *donovani* promastigotes were added to THP1 culture for 4 hours at a ratio of 1:10 macrophage to parasite. Subsequently, plates were washed three times with room temperature PBS and then incubated for 48 hours in RPMI 1640 medium. Macrophages, were collected with a scraper and washed with ice-cold PBS for 4 minutes at 48Xg. The pellets were then resuspended in 1 ml PBS and transferred to microcentrifuge tubes. Separation of amastigotes was facilitated initially by passing the pellet repeatedly through a 1 ml syringe with a 27G needle. Lysates were then centrifuged for 15 minutes at 2500Xg at 4°C. The pellet was then resuspended in 45% Percoll which built the middle layer of the Percoll gradient (containing from bottom to top; 1.5 ml of 75%, 3 ml of 45% and 5.5 ml of 25%). Cells were then centrifuged at 3500Xg for 60 minutes at 4°C as previously described [51].

### Arginine deprivation

Mid log phase promastigotes or amastigotes (1×10^7^ cells/ml) were washed twice in Earl’s salt solution and resuspended to a final density of 1×10^7^ cells/ml in amino acid deficient medium 199 Earl's salt base (Biological Industries Ltd., Beit Haemek, Israel). This deprivation medium was supplemented with 10% heat-inactivated, dialyzed (10,000 kDa cutoff) fetal calf serum and all amino acids except arginine. Deprivation was carried out at 26°C for times indicated in each experiment, and terminated by moving the cells to ice. Deprived cells were subjected to transport assays, western and northern analyses after two additional washes in ice cold Earl's salt solution.

### Transport assays

Uptake of 25 μM L-[H^3^] arginine (600 mCi/mmol) into mid log-phase *L*. *donovani* was determined using the rapid filtration technique previously described for axenic promastigotes and amastigotes [52]. Briefly, transport reaction mixture contained 1×10^8^/ml axenic amastigotes or promastigotes suspended in Earl's salt solution at either pH 7 (for promastigotes) or 5.5 (for amastigotes), supplemented with 5 mM glucose and 1% dialyzed fetal calf serum (heat-inactivated for promastigotes only). Cells, were pre-warmed at 30°C (promastigotes) or 37°C (amastigotes) for 10 min prior to addition of radiolabeled arginine. At times indicated in each experiment, cell suspensions were subjected to rapid filtration as described previously [21]. The amount of radiolabel associated with the cells was linear with time over the 3-min time course of the transport assay.

### Proteomic analysis of arginine deprivation response

#### a) Protein extraction

Promastigotes were deprived from arginine for 5 or 15 minutes. Subsequently, cell were lysed in 1 ml lysis buffer (containing 8 M urea, 75mM NaCl, 50mM Tris-Cl at pH 8.2, 1mM NaF, 1mM β -glycerophosphate, 1mM Sodium Orthovandate, 10mM Sodium Pyrophosphate and 1mM PMSF), then vortexed and sonicated. Proteins in solution were reduced using 2.8 mM dithiothreitol (DTT, 60μC for 30 min), modified with 8.8 mM iodoacetamide in 100 mM ammonium bicarbonate (in the dark, room temperature for 30 min) and finally digested in 2 M urea, 25mM ammonium bicabonate with modified trypsin (Promega Inc., USA) at a 1:50 enzyme-to-substrate ratio, overnight at 37°C. Subsequent second digestion was carried out for 4 hours.

#### b) Dimethyl labeling

The resulting peptides were desalted using C18 Sepak columns (Waters Inc., USA) dried and re-suspended in 50 mM HEPES (pH 6.4). Labeling using dimethylation technique was carried out in the presence of 100 mM NaCBH_3_ (Sterogene Ltd., USA), by adding light formaldehyde (35% w/v of 12.3 M, Frutarum Ltd., USA) to one of the samples, and heavy formaldehyde (20% w/w, of 6.5 M, Cambridge Isotope Laboratories, USA) to the other sample to a final concentration of 200mM. After 1 hour incubation at room temperature the pH were raised to 8 and incubation continued for additional 1 hour. Neutralization was done using 25mM ABC for 30 min and equal amounts of the light and heavy peptides were mixed, cleaned on C18 Sepak columns and re-suspended in 0.1% formic acid.

#### c) Phosphoprotein enrichment

Peptides were separated using a strong cation exchange column (SCX). five μg of each fraction were kept for MS analyses. Phosphopeptide enrichment was carried out using titanium dioxide (TiO2) column.

#### d) Mass spectrometry analysis

Each SCX fraction of the phospho-enriched peptides was resolved by reverse-phase chromatography on 0.075 X 200 mm fused silica capillaries packed with Reprosil reversed phase material (Dr Maisch GmbH, Germany). Peptides were eluted using linear 214 minute gradients of 7 to 40% and 8 minutes at 95% acetonitrile with 0.1% formic acid in water at a flow rate of 0.25 μl/min. Mass spectrometry performed in an ion-trap Orbitrap mass spectrometer (Thermo Inc., USA). Positive mode employed using repetitively full MS scan followed by collision induces dissociation (CID Ltd., USA) of the 10 most dominant ion selected from the first MS scan. In order to analyze phosphopeptides multistage activation was used. MS data analyzed using the Proteome Discoverer 1.3 (Thermo Inc., USA) software searching against the *L*. *infantum* proteome in TriTrypDB v4 (http://tritrypdb.org/tritrypdb/). Results were filtered with rank 1 peptides and 1% false discovery rate. The ratios normalized according to proteins median ratio. Perseus (Mathias Mann's laboratory, Germany) software was used for data statistical analysis. The results were further filtered according to a pRS probability >0.65 and valid values. Significance B was calculated with p-value for detection of significant outlier ratios, as described in [53]. The mass spectrometry proteomics data have been deposited to the ProteomeXchange Consortium [54] via the PRIDE partner repository with the dataset identifier PXD002830.

### RNAseq analysis

Total RNA was isolated from two biological replicates of *L*. *donovani* promastigotes grown for 15 minutes with or without arginine; axenic amastigotes grown with or without arginine for 2 hours; and amastigotes obtained 48 hours after infection of THP1 macrophages with late log-phase promastigotes. RNA-seq libraries were prepared by Genewiz (South Plainfield, NJ, USA) using a NEBNext Ultra RNA Library Preparation Kit according to the manufacturer’s instructions for the Poly(A) mRNA Magnetic Isolation Module. Individual libraries were run on an Agilent 2100 Bioanalyzer to check insert size before being quantitated using Qubit and pooled. The pooled libraries were quantified using qPCR following KAPA SYBR Fast Universal qPCR kit according to manufacturer’s instructions, and clustering on a flowcell for sequencing on an Illumina HiSeq2500 machine in the 2x50bp PE Rapid Run Mode according to manufacturer’s instructions. Reads from each library were mapped against the *L*. *infantum* JCPM5 genome in TriTrypDB v7 (since it is currently better assembled than *L*. *donovani* BPK281A1) using BOWTIE2 and normalized reads counts for each CDS determined using EdgeR. The normalized read counts from each sample were compared to those obtained for promastigotes grown in complete medium to calculate an average log2 fold change for each protein-coding gene. The RNA-seq data have been submitted to the NCBI GEO database with accession number GSE71572 and will also available in a future release of TriTrypDB.

### Subcellular fractionation

Mid log-phase 1×10^9^ *L*. *donovani* promastigotes at a density of 1×10^7^ cells/ml were resuspended in hypotonic lysis buffer (5mM HEPES pH 7.4, 2 mM EGTA, 2 mM DTT and protease inhibitor cocktail) and lysed with 20 passes through a 27 gauge needle. Lysates then shifted to isotonic solution by adding 0.25 volumes of a solution containing 50 mM HEPES pH 7.4, 0.25M sucrose, 1mM ATP, 1mM EGTA, protease inhibitor cocktail. Subsequently, cells centrifuged at 3000×g for 15 min at 4°C. Post-nuclear lysate (5.0 ml) was layered over a 8.0 ml 20–60% linear ultrapure sucrose gradient with a 70% cushion. Samples subjected to centrifugation at 218,000×g for 6 h at 4°C in a Beckman SW41 rotor [55]. Gradients were fractionated from the top (500 μl/fraction) and Laemmli sample buffer was added prior to Western blot analysis using anti-LdPEX14, anti-LdAAP3 and anti-LdAAP24 antibodies.

A crude organelle fraction containing glycosomes was prepared by centrifugating *L*. *donovani* postnuclear supernatants at 45,000×g for 60 min at 4°C. The pellet was then resuspended in PBS and Laemmli sample buffer.

### Determination of cellular arginine content

Cellular arginine was determined using standard clinical chemistry techniques as described previously [21, 27]. Intracellular arginine concentrations were calculated based on a previously determined promastigote cell volume of 4.2 μl per 10^8^ cells [56].

### Immunofluorescence

Indirect immunofluorescence analysis was carried out following the methods described in Inbar et al. [27]. Briefly, mid-log phase promastigotes were washed twice in PBS and then fixed in 1% formaldehyde/PBS on a slide for 10 min before permeabilization by exposure to 0.2% TritonX-100/PBS for 10 min. Cells were incubated with blocking solution [10% (v/v) non-fat dried skimmed milk powder/PBST] for 30 min at room temperature, incubated with anti-LdAAP3 antibodies (1:500 dilution) for 1 h and then incubated with secondary polyclonal goat anti-rabbit IgG fluorescent antibodies (1:500 dilution; Dy-light 549 Red; Jackson) for 1 h in the dark at room temperature. Finally, cells were washed in PBST and supplemented with 5 μl DAPI (4',6-diamidino-2-phenylindole; 0.5 μg/ml; Fluka). Fluorescence analyses were carried out using a fluorescent microscope (Axiovert 200M; Zeiss).

### Western blot analyses

Western blot analysis of LdAAP3 was done as described previously [21].

### Northern blot analyses

Total RNA from *L*. *donovani* promastigotes (either starved for arginine or non- starved) was prepared and subjected to Northern blotting of *LdAAP3* as described before [21]. Probes were amplified using the following primers: LinJ.31.900 AAP3 Forward: 5’-ATCATGAATTCATGAGCAAGCCCAGCAAGT-3’. Reverse: 5’-GCTTAGTCGACCGGAAGATGATGTTGCGC-3’.

## Supporting Information

S1 Table Phosphorylation changes following 5 minute of arginine deprivation in *L*. *donovani* promastigotes. Di methylation of tryptic peptides extracted from *L*. *donovani* promastigotes 5 min after arginine deprivation initiated. Phosphopeptide enrichment, mass spectrometry and data analysis is described in detail in Materials and Methods.
(PDF)

S2 Table Phosphorylation changes following 15 minute of arginine deprivation in *L*. *donovani* promastigotes. Di methylation of tryptic peptides extracted from *L*. *donovani* promastigotes 15 min after arginine deprivation initiated. Phosphopeptide enrichment, mass spectrometry and data analysis is described in detail in Materials and Methods.
(PDF)
