# Supplementary material for: An Arginine Deprivation Response Pathway Is Induced in Leishmania during Macrophage Invasion
Source: PLoS Pathog. 2016 Apr 4;12(4):e1005494. doi: 10.1371/journal.ppat.1005494 (PMC4846328; doi:10.1371/journal.ppat.1005494)
Supplement: S1 Table — Di methylation of tryptic peptides extracted from L. donovani promastigotes 5 min after arginine deprivation initiated. Phosphopeptide enrichment, mass spectrometry and data analysis is described in detail in Materials and Methods. (PDF) [file ppat.1005494.s001.pdf]

Supplements

Table S1: Phosphorylation changes following 5 minute of arginine deprivation in *L. donovani* promastigotes

**Accession number**	**Description**	**Phosphorylated tryptic peptide**	**Repeat 1**	**Repeat 2**
			Log$_2$ fold from non-starved	
LinJ.27.1120	histone H1, putative	SSDSAVAALSAAMT**p**SPQK	2.537	2.51
LinJ.33.1330	hypothetical protein, conserved	EA**p**SAVAPPPLLHHFTTQQEPK	1.809	1.09
LinJ.26.2010	hypothetical protein, conserved	KVHAGDNEGDAGA**p**SAPR	1.47	1.13
LinJ.19.0580	hypothetical protein, conserved	IQLAAGL**p**SR	7.59	1.1
LinJ.22.0040	hypothetical protein, conserved	G**p**SFSHNPYAEAS**p**SFQN	6.55	2.85
LinJ.27.1080	hypothetical protein, conserved	RK**p**SDNAVTLSSSSSVR	4.28	1.09
LinJ.34.4190	RNA-binding protein-like protein	HWLP**p**SPQYR	4.23	3.09
LinJ.20.0650	hypothetical protein	RP**p**SAPSLAPPR	2.93	1.75
LinJ.34.2150	hypothetical protein, unknown function	LAN**p**SFSNASCHLER	2.43	3.03
LinJ.32.1190	hypothetical protein, conserved	SGPSA**p**SPTAK	2.29	1.12
LinJ.06.1010	hypothetical protein, conserved	SL**p**STLQK	2.14	1.75
LinJ.11.0400	tubulin-tyrsoine ligase-like protein	H**p**SVVGAEAASAASK	2.02	1.91

LinJ.25.2450	serine/threonine-protein kinase, putative	LFGGF**p**SCTADSHLNNS	2	2.99
LinJ.13.1350	MCAK-like kinesin, putative	DPNTDDGENAVRPAAAPAKPV**p**SRPV**p**SR	1.96	1.38
LinJ.22.0040	hypothetical protein, conserved	ASR**p**SPLICSQR	1.96	2.04
LinJ.33.2270	hypothetical protein, conserved	ESVSAA**p**SPNATVAASR	1.96	1.22
LinJ.35.0320	serine palmitoyltransferase, putative	EIALQEASDAVDNKSQVL**p**SK	1.96	1.97
LinJ.36.2040	inosine-guanosine transporter	AVP**p**SDAFVAK	1.89	1.32
LinJ.36.1720	universal minicircle sequence binding protein (UMSBP), putative	GR**p**SGAQGGYGGDR	1.79	1.45
LinJ.13.1350	MCAK-like kinesin, putative	SYHRPTP**p**TH**pS**R	1.69	0.92
LinJ.31.2130	hypothetical protein, conserved	NN**p**SFFSK	1.58	2.07
LinJ.11.0400	tubulin-tyrsoine ligase-like protein	APATAESASNQPHQRH**p**SVVGAEAASAASK	1.53	1.47
LinJ.09.0520	serine/threonine protein phosphatase, putative	DAD**p**TPVVSSK	1.44	0.99
LinJ.06.1020	hypothetical protein, conserved	SSVSV**p**TPSR	1.4	2.18
LinJ.33.1090	hypothetical protein, conserved	SGKPGASAATAS**p**SPIGASANGAR	1.38	1
LinJ.34.2390	hypothetical protein, conserved	VREP**p**SAANTR	1.35	0.94
LinJ.10.0320	hypothetical protein, conserved	RGP**p**SAGDNVAQDVIVITSGDDLLR	1.34	0.99
LinJ.16.1550	kinesin, putative	GHPVGVA**p**TPK	1.34	0.81

LinJ.24.1000	hypothetical protein, conserved	RG**p**SLGGANAQQR	1.33	1.49*
LinJ.30.1280	hypothetical protein, unknown function	KQHNVD**p**TPSSF**p**SLAGEEGGTAK	1.31	1.15
LinJ.11.0520	nucleobase transporter	KAELLLETEHGDV**p**TAR	1.3	1.55
LinJ.19.0690	kinesin, putative	SD**p**SISA**p**TPTR	1.3	1.03
LinJ.21.1850	hypothetical protein, conserved	VEQVLAHQMSP**p**SASGR	1.3	1.74
LinJ.01.0790	eukaryotic initiation factor 4a, putative	FCETFVGG**p**TR	1.29	2.12
LinJ.34.0640	hypothetical protein, conserved	RL**p**SLHLE**p**SPLK	1.25	1.02
LinJ.07.0780	topoisomerase-related function protein	ALDADALQATVHLQP**p**SPSHGEK	1.19	1.36
LinJ.34.2680	PKA regulatory subunit of protein kinase a-R'	TIQMQRP**p**SR	1.19	1.06
LinJ.12.0610	serine/threonine protein phosphatase-like protein	RTY**pS**NG**p**STEVK	1.18	1.05
LinJ.19.0290	RNA binding protein, putative	AGNV**p**SINQHEGQR	1.18	1.03
LinJ.28.1130	hypothetical protein, unknown function	VA**p**SAPPALAVTGK	1.17	1.25
LinJ.35.2610	hypothetical protein, conserved	RPSP**p**SLNDPPAFVR	1.16	0.95
LinJ.05.0850	hypothetical protein, conserved	**p**SRHV**p**TPGHGSADSGSSPGK	1.13	1.5

LinJ.12.0610	serine/threonine protein phosphatase-like protein	NT**p**SFSGSGTGR	1.13	1.14
LinJ.13.1350	MCAK-like kinesin, putative	MVN**p**SFHR	1.12	1.11
LinJ.15.1170	hypothetical protein, conserved	RS**p**SEGSPIR	1.11	0.94
LinJ.36.0780	MPK2 mitogen-activated protein kinase	KT**p**SVS**p**SASAGGSR	1.1	0.99
LinJ.32.0320	hypothetical protein, conserved	RS**p**SVIDEVDR	1.07	1.2
LinJ.10.0110	hypothetical protein, conserved	NAVDDLF**p**SDDDKPK	1.06	0.89
LinJ.35.3250	hypothetical protein, conserved	EFVPHFQLIAN**p**SPEYRPTPPPK	1.06	1.45
LinJ.20.1130	hypothetical protein, conserved	GTAA**p**SFTDHSR	0.94	0.79
LinJ.32.1190	hypothetical protein, conserved	FTPI**p**SPSVASPATKR	0.91	0.95
LinJ.11.0140	hypothetical protein, conserved	GQLNYDHLLDEVV**p**SK	0.9	2.87
LinJ.28.2120	hypothetical protein, conserved	AVSPAEQEQALR**p**SQC**p**S**p**TPR	0.9	1.03
LinJ.19.1080	hypothetical protein, conserved	AEHRA**p**SLPALTAVLD**p**SPVK	0.88	0.77
LinJ.24.2010	hypothetical protein, conserved	AT**p**SEVLAHPAR	0.88	0.86
LinJ.24.1000	hypothetical protein, conserved	RA**p**SQHLAAPSPR	0.85	1.28
LinJ.30.3130	hypothetical protein, conserved	KV**p**SEAVGDR	0.82	0.89
LinJ.32.0270	serine/threonine-protein kinase Nek1, putative	RGAPPGEPA**p**SRP**p**TTPQQQR	0.8	0.75

LinJ.34.0640	hypothetical protein, conserved	RH**p**SNFDLSVGTPLPLGGSGK	0.8	1.18
LinJ.11.0400	tubulin-tyrsoine ligase-like protein	VRPPLLGPLLTT**p**SR	0.79	0.59
LinJ.13.1350	MCAK-like kinesin, putative	VGAS**p**SP**p**SPFGR	0.79	0.44
LinJ.04.0060	hypothetical protein, conserved	AGHFYGA**p**SVR	0.78	0.71
LinJ.09.0840	hypothetical protein, conserved	APP**p**TPSQQK	0.75	1.09
LinJ.08.0130	hypothetical protein, conserved	AAQASPFAGQN**p**SR	0.74	0.93
LinJ.08.0130	hypothetical protein, conserved	VVVVESEAP**p**SPHARP**p**SGHAAFQR	0.74	0.81
LinJ.13.0700	hypothetical protein, conserved	GDNLLHDSDQTPCDAAAAGSSIVANN**p**TPR	0.72	0.83
LinJ.16.0390	tubulin tyrosine ligase, putative	VDEAPL**p**TSR	0.7	0.92
LinJ.36.7250	hypothetical protein, conserved	VGLPIFGHAFQFKPVTESAE**p**SPR	0.7	1.02
LinJ.35.4190	hypothetical protein, conserved	SR**p**SVNTTQGSVPGAKPVPSR	0.69	0.83
LinJ.36.0780	Mitogen-activated protein kinase 2 (MPK2)	SILSLEGEQASRPVL**p**TD**p**YIATR	0.69	0.78
LinJ.27.0630	hypothetical protein, conserved	LRPV**p**SQHGAK	0.51	0.94
LinJ.28.2120	hypothetical protein, conserved	AV**p**SPAEQEQALR**p**SQC**p**S**p**TPR	0.41	1.03
LinJ.20.1130	hypothetical protein, conserved	AAFRPLG**p**SSDTEDVDDFTAPSR	-0.48	-2.19
LinJ.34.3220	DNA topoisomerase IB, large subunit	ANKVV**p**SDDDDVPLAALRV	-0.62	-0.67
LinJ.05.0870	hypothetical protein, conserved	GASAPTLPS**p**SPR	-0.67	-3.62

LinJ.36.4540	hypothetical protein, conserved	YLGGANNIHSYVG**p**SPGQSFR	-0.68	-0.76
LinJ.27.0550	hypothetical protein, conserved	DSMVVEELKADAIN**p**SGGR	-0.72	-0.66
LinJ.22.0060	hypothetical protein, conserved	ASVENARH**p**SAQNAVDNAK	-0.74	-1.11
LinJ.36.6110	hypothetical protein, conserved	KRGG**p**SINGATALLR	-0.75	-0.69
LinJ.31.2870	hypothetical, protein kinase	SHTHTQLEESEMPNA**p**TPK	-0.76	-1.07
LinJ.20.1130	hypothetical protein, conserved	AAFRPLG**p**S**p**SD**p**TEDVDDFTAPSR	-0.77	-2.19
LinJ.28.2710	hypothetical protein, conserved	HPQPTVQPTPS**p**SPALDVAEGLHFQK	-0.77	-0.9
LinJ.10.0200	MPK10 mitogen-activated protein kinase	TH**p**SLMELAGNAPAPS	-0.83	-0.65
LinJ.11.0880	hypothetical protein, conserved	TVAAPHKAD**p**STASPTAPEIVK	-0.84	-0.81
LinJ.21.0700	phosphoglucomutase, putative	KATGAFILTA**p**SHNPGGPDADFGIK	-0.85	-0.51
LinJ.28.2710	hypothetical protein, conserved	HPQPTVQPTP**p**SSPALDVAEGLHFQK	-0.86	-0.9
LinJ.17.0310	hypothetical protein, conserved	QSRPSSVSLLADDRAPP**p**SPVHEPLLGVAAEEEANR	-0.87	-1.1
LinJ.17.0490	hypothetical protein, conserved	AAGGQQTTPH**p**SPV**p**SPGKK	-0.87	-1.37
LinJ.20.1140	phosphatidylinositol 3-kinase, putative	SL**p**SGPTPNNVVSGNR	-0.9	-6.45
LinJ.31.1410	hypothetical protein, unknown function	TEEEADAVHH**p**SAASER	-0.91	-1.65
LinJ.32.1290	ADP-ribosylation factor GTPase activating protein 1, putative	VAVQAKPVGPA**p**SPE**p**SSPVCQGR	-0.99	-0.74

LinJ.35.0390	hypothetical protein, conserved	AL**p**SEDKDGVWTPVAR	-1	-0.87
LinJ.17.1000	hypothetical protein, conserved	FTTTARP**p**SVA**p**TVAGEDVSTLQHR	-1.01	-0.8
LinJ.28.2120	hypothetical protein, conserved	HRHGESAPTDPAAATSAGALLR**p**TPR	-1.04	-6.64
LinJ.29.1270	hypothetical protein, conserved	SFCSFAHADADGGNA**p**SEEGER	-1.07	-1.09
LinJ.11.0140	hypothetical protein, conserved	AP**p**S**p**TPPEDCNGPCQVLVEFK	-1.13	-2.16
LinJ.35.2530	hypothetical protein, conserved	AG**p**S**p**SIADTPARPWPEDAGLSLKR	-1.15	-1.2
LinJ.25.1100	hypothetical protein, conserved	EEP**p**SEGHRDDKVAESVATISIR	-1.16	-1.32
LinJ.28.2370	glycoprotein 96-92, putative	**p**SVATVEAAKK	-1.24	-2.32
LinJ.27.1720	hypothetical protein, unknown function	SAPS**p**SPSNVPGR	-1.28	-1.43
LinJ.20.0750	hypothetical protein, conserved	GDD**p**SEDREH**p**SDVDDDGHKSGAAGTVADVAAAEK	-1.34	-0.86
LinJ.31.1400	hypothetical protein, unknown function	EAP**p**SPSVGASLVD**p**SDRTGAAR	-1.36	-0.84
LinJ.25.1100	hypothetical protein, conserved	EEP**p**SEGHRDDKVAE**p**SVATI**p**SIR	-1.4	-1.32
LinJ.36.4790	hypothetical protein, conserved	SHHDSLMA**p**SPQATQK	-1.46	-1.53
LinJ.26.0790	asparagine synthetase a, putative, aspartate--ammonia ligase, putative	APDYDDW**p**SSPVSVDSSK	-1.5	-1.02
LinJ.01.0480	hypothetical protein, conserved	AVAESAPS**p**SPTTGK	-1.57	-0.87
LinJ.29.1510	RNA binding protein, putative	KG**pT**ASPAASVPHR	-1.58	-1.47

LinJ.29.1510	RNA binding protein, putative	KGTA**p**SPAASVPHR	-1.58	-1.47
LinJ.30.0460	eukaryotic translation initiation factor 4e, putative	SLQN**pS**PIIQPSR	-1.59	-0.69
LinJ.27.1720	hypothetical protein, unknown function	SAPS**pS**PSNVPGRK	-1.61	-1.35
LinJ.34.1390	hypothetical protein, conserved	LLGAAEAH**pS**PTIWR	-1.84	-1.22
LinJ.35.4450	hypothetical protein, conserved	RP**pS**PKP**pS**PK	-1.96	-1.26
LinJ.10.1010	hypothetical protein, conserved	AP**pS**GGLSAEGWGGR	-2.07	-1.27
LinJ.21.0880	hypothetical protein, conserved	QPG**pS**PNKALAAIDPAIVSAASSQSK	-2.11	-1.51
LinJ.29.1510	RNA binding protein, putative	KD**p**TPLLLPR	-2.29	-2.21
LinJ.32.1420	RNA guanylyltransferase, putative	SLAGAGAV**pS**NDDVPLLSGFKR	-2.57	-2.27
LinJ.03.0910	hypothetical protein	TAAMNRPYGYNVT**pT**EGDID**pS**DLDDAAISAILK	-2.59	-2.01
LinJ.04.0520	hypothetical protein, conserved	GAAVDLFAASPLAS**pS**PTDNQQR	-2.64	-1.06
LinJ.21.0770	ATP-binding cassette protein subfamily E, member 1, putative	NGDD**pS**DNDDGSDVKAAPK	-2.64	-1.14
LinJ.30.2890	hypothetical protein, conserved	GVVS**pS**PTSAAPASAR	-3.51	-0.39
LinJ.35.5360	polyadenylate-binding protein 1, putative	HSPGDVPE**pT**PPLPPITPQELESMSPQEQR	-6.65	-6.66

Di methylation of tryptic peptides extracted from *L. donovani* promastigotes 5 min after arginine deprivation initiated. Phosphopeptide enrichment, mass spectrometry and data analysis is described in detail in Experimental Procedures
