# Supplementary material for: An Arginine Deprivation Response Pathway Is Induced in Leishmania during Macrophage Invasion
Source: PLoS Pathog. 2016 Apr 4;12(4):e1005494. doi: 10.1371/journal.ppat.1005494 (PMC4846328; doi:10.1371/journal.ppat.1005494)
Supplement: S2 Table — Di methylation of tryptic peptides extracted from L. donovani promastigotes 15 min after arginine deprivation initiated. Phosphopeptide enrichment, mass spectrometry and data analysis is described in detail in Materials and Methods. (PDF) [file ppat.1005494.s002.pdf]

Table S2: Phosphorylation changes following 15 minute of arginine deprivation in *L. donovani* promastigotes

**Accession number**	**Description**	**Phosphorylated tryptic peptide**	**Repeat 1**	**Repeat 2**
			Log_2 fold from non-starved	
LinJ.27.1700	protein kinase-like protein	G**p**SPDVDDKFVDVGDATK	6.67	1.68
LinJ.24.0160	hypothetical protein, conserved	GGEEEGTNFIVD**pS**DDEDEAVAAIR	6.6	1.9
LinJ.15.0810	protein kinase, putative	APPVSYNEPGHEQLD**pS**R	6.55	1.73
LinJ.13.1330	hypothetical protein, conserved	LSYSAATAE**pS**PSK	5.48	1.55
LinJ.35.2530	hypothetical protein, conserved	AG**pSpS**IADTPARPWPEDAGLSLK	5.05	2.06
LinJ.35.4450	hypothetical protein, conserved	APIAD**pS**DS**p**DDDEPVR	4.64	3.31
LinJ.08.0630	hypothetical protein, conserved	ASS**pT**EGSPVAAGTIPAVAAK	4.28	2.67
LinJ.30.0520	hypothetical protein, conserved	FA**pS**PDKLETVTPTK	4.25	0.95
LinJ.22.1070	hypothetical protein, conserved	**pS**LDGAAGDAEECADAASASTGFPR	3.34	1.36
LinJ.09.0840	hypothetical protein, conserved	**pS**PLATPTAVFSPSNSK	2.76	6.74
LinJ.34.3220	DNA topoisomerase IB, large subunit	VV**pS**DDDDVPLAALR	2.08	1.62
LinJ.27.1100	hypothetical protein, conserved	V**pS**NGQTIMQLQDELETLHR	2.02	1.12
LinJ.31.0450	cytoskeleton-associated protein CAP5.5, putative,	VPQPEAQ**pS**AKV**pSpS**ETA**pS**VEEQR	1.84	1.75
LinJ.35.3150	ATP-dependent RNA helicase, putative	GGG**pT**GNEDDDYEDGGYDAYR	1.77	1.92
LinJ.35.4450	hypothetical protein, conserved	APIAD**pS**D**pS**DDDEPVRKPVLAK	1.66	1.29
LinJ.36.5290	hypothetical protein, conserved	VQVMTEDMNEDG**pS**NDER	1.63	1.48
LinJ.36.2260	DEAD box RNA helicase, putative	FDpY**SpS**DDDDSARPAK	1.61	1.29
LinJ.01.0790	eukaryotic initiation factor 4a, putative	FCETFVGG**pT**R	1.59	1.88
LinJ.36.6110	hypothetical protein, conserved	KC**pS**APELPPR	1.57	3.48
LinJ.35.0920	hypothetical protein, conserved	LLNNENADDLL**pSpS**DDEEPK	1.48	1.91
LinJ.25.1100	hypothetical protein, conserved	F**pS**IEGNEPEQKPLVPTSHLR	1.39	2.48

LinJ.13.0700	hypothetical protein, conserved	GDNLLHDSDQTPCDAAAAGSSIVANN**pT**PR	1.37	1.23
LinJ.11.1110	60S ribosomal protein L28, putative	AVAAVRPDLADV**pS**FRR	1.36	1.19
LinJ.14.1300	hypothetical protein, conserved	AV**pS**VVDGPHGPHAR	1.34	2.14
LinJ.35.2240	RNA-binding protein, putative	H**pS**HG**pS**NDEIQNGGDTDNFR	1.29	3.4
LinJ.31.2440	hypothetical protein, conserved	GNTD**pS**PDTAELAAAALQALSPQGVLHTDSR	1.26	1.73
LinJ.35.2610	hypothetical protein, conserved	TNTSATVT**pS**EGNSNSEK	1.24	0.77
LinJ.08.1040	Phosphoribosylpyrophosphate synthetase	MTEVHAMSGTV**pS**PHTEK	1.22	0.99
LinJ.35.4450	hypothetical protein, conserved	APIAD**pS**D**pS**DDDEPVRKPVAK	1.22	1.1
LinJ.35.4060	protein kinase A catalytic subunit isoform 1	SPGDTSNFEKYPD**pS**PVDR	1.19	1.13
LinJ.31.2700	serine/threonine protein phosphatase pp1(5.9), putative	DAN**pS**PLSSFAR	1.16	1.68
LinJ.27.1630	hypothetical protein, conserved	NLP**pS**VDDGLYQK	1.14	1.46
LinJ.04.0060	hypothetical protein, conserved	GNEEEERNE**pT**PRDPCK	1.01	1.18
LinJ.01.0480	hypothetical protein, conserved	AVAESAPS**pS**PTTGK	0.98	0.9
LinJ.33.2570	hypothetical protein, conserved	Q**pS**ATATTAAPGEDVDPMEAK	0.97	0.95
LinJ.33.2570	hypothetical protein, conserved	Q**pS**ATATTAAPGEDVDPMEAK	0.97	0.89
LinJ.36.2490	tyrosine aminotransferase	AGNSTG**pS**LTEER	0.94	1.25
LinJ.36.2490	tyrosine aminotransferase	AGN**pS**TGSLTEER	0.94	1.1
LinJ.31.0090	hypothetical protein, conserved	**pS**AEKAPEKPVADKEAEDAEK	0.68	0.78
LinJ.11.0150	hypothetical protein, conserved	IQDVNEDSGLPFDVE**pS**AHREGD**pS**PAR	0.55	1.3
LinJ.34.0500	calcium channel protein, putative,ion transporter, putative	LYRQP**pS**LPATEMGF**pS**VVGTPQQR	-0.81	-1.94
LinJ.13.1350	MCAK-like kinesin, putative	ERVAEQPRPM**pS**AQR	-0.85	-3.31
LinJ.20.1130	hypothetical protein, conserved	GYEG**pT**D**pS**EDGENHHHSAAGAGKK	-0.85	-0.63
LinJ.35.4450	hypothetical protein, conserved	RP**pS**PKP**pS**PK	-0.9	-3.01
LinJ.32.0370	hypothetical protein, conserved	SAAAGAG**pS**AKRPAGLSPGAAAALAADEK	-0.92	-0.93
LinJ.36.1560	hypothetical protein, conserved	AGKQH**pS**DEEVELSVK	-0.94	-1.71

LinJ.19.1140	hypothetical protein, conserved	GDD**pS**EDREH**pS**DVDDDGHKSGAAGTVADVAAAEK	-1.03	-1.78
LinJ.19.1140	hypothetical protein, conserved	GDDSEDREH**pS**DVDDDGHKSGAAGTVADVAAAEK	-1.03	-2.24
LinJ.13.0290	flagellar radial spoke protein, putative	ITSSTTIAPQGALEEPEPEQE**pT**EEDEDEDAEDGAQRKPK	-1.05	-6.65
LinJ.19.1140	hypothetical protein, conserved	QRHT**pS**PSPYEQVPK	-1.2	-1.94
LinJ.29.1510	RNA binding protein, putative	D**pT**PLLLPR	-1.29	-1.22
LinJ.29.2540	hypothetical protein, unknown function	RYVPPSEEQDAEAAGQ**pS**ASR	-1.5	-4.52
LinJ.21.0920	hypothetical protein, conserved	AR**pS**QLAAVRDDLAQR	-1.52	-2.51
LinJ.32.2950	RNA-binding protein, putative	AR**pS**GEDDCDDEENTFNFKR	-1.59	-6.7
LinJ.30.0580	hypothetical protein, conserved	TTGRDPNED**pS**G**pS**DAEEVTEEELAARK	-1.61	-4.87
LinJ.25.1920	hypothetical protein, conserved	AKPVVPFDVSDYEDKKVQ**pS**PK	-1.65	-2.61
LinJ.33.3230	hypothetical protein, conserved	VG**pT**PVEPHRGALEGA**pS**PVDAR	-1.72	-3.42
LinJ.33.3230	hypothetical protein, conserved	VG**pT**PVEPHRGALEGASPVDAR	-1.72	-6.33
LinJ.12.0720	hypothetical protein, conserved	DLLAR**pT**PDVGDDDDEDLRR	-1.77	-1.63
LinJ.22.0060	hypothetical protein, conserved	ASVENARH**pS**AQNAVDNAK	-1.78	-1.29
LinJ.16.1550	kinesin, putative	SV**pS**GTG**pS**KGTFKR	-1.8	-1.37
LinJ.18.1510	P-type H^+-ATPase, putative	KYELDAAAFEDKPE**pS**H**pS**DAEMTPQKPQRR	-1.8	-2.79
LinJ.05.0140	nucleolar RNA helicase II, putative	ISAKA**pS**DHGNGVAVEK	-1.81	-2.03
LinJ.20.1670	hypothetical protein, conserved	EQRA**pS**PATVVAPEDKK	-1.86	-5.88
LinJ.34.0700	hypothetical protein, conserved	AAEPELADADGVEPSSGPTSSR**pS**PTGR	-1.88	-1.23
LinJ.36.6780	ubiquitin fusion degradation protein, putative	VAAG**pT**PPLKHLPGQSSSPSSGPK	-1.95	-6.75
LinJ.15.0300	hypothetical protein, conserved	AALPEALAVHAHDWD**pS**SSSGRGR	-1.97	-1.95
LinJ.11.0520	nucleobase transporter	YLADSG**pS**EKKAELLLETEHGDVTAR	-1.99	-1.34
LinJ.29.0280	hypothetical protein, conserved	LAAR**pS**PAK**pS**PAR	-2	-1.44
LinJ.17.1310	hypothetical protein, unknown function	VFNRDG**pS**AVAQR	-2.07	-2.77
LinJ.21.1850	hypothetical protein, conserved	MRVEQVLAHQM**pS**PSASGR	-2.09	-1.63

LinJ.36.6110	hypothetical protein, conserved	KRGG**pS**INGATALLR	-2.17	-6.7
LinJ.27.1220	hypothetical protein, conserved	HVF**pT**PEGCKYGDK	-2.27	-6.75
LinJ.29.0120	proteasome regulatory non-ATPase subunit, putative	EGNA**pS**EEAAAKPEEEKTAPLNHK	-2.36	-1.57
LinJ.34.3220	DNA topoisomerase IB, large subunit	ANKVV**pS**DDDDVPLAALRV	-2.39	-2.74
LinJ.04.0060	hypothetical protein, conserved	NE**pT**PRDPCKL**pT**DVSTDHYAK	-2.4	-6.67
LinJ.21.0920	hypothetical protein, conserved	AL**pS**AV**pS**EKNADFNDLRR	-2.65	-1.21
LinJ.05.0100	phosphoprotein phosphatase, putative	ETKGPV**pS**PR	-2.7	-2.6
LinJ.04.0060	hypothetical protein, conserved	VNIDNVERHPPVPEGVN**pT**PR	-2.87	-6.67
LinJ.34.1610	hypothetical protein, conserved	DVGDHREA**pS**VL**pT**AAGR	-2.93	-2.37
LinJ.14.0860	hypothetical protein, conserved	KASAGGDDDEAQ**pS**PESSPKETK	-3.05	-6.73
LinJ.36.6290	hypothetical protein, conserved	EAVEHA**pS**VQDTAAKEVEATK	-3.13	-3.63
LinJ.24.2180	hypothetical protein, conserved	AHKDYVVPELPEEPMWPLIGH**pT**P	-3.17	-1.52
LinJ.18.1510	P-type H -ATPase, putative	ALEGLREDTHVLNEST**pS**PVNAFSPK	-3.28	-1.74
LinJ.31.1230	hypothetical protein, conserved	AER**pS**PFGGGFECPEGYWVCSVEHGGCSK	-4.6	-4.91
LinJ.29.0280	hypothetical protein, conserved	SPAK**pS**PARAN**pS**PEPQPMTSASSTPR	-4.72	-6.38
LinJ.13.1350	MCAK-like kinesin, putative	VQAAIQAPALPARIP**pT**PLRR	-4.76	-4.32
LinJ.07.0620	hypothetical protein, conserved	KAAAAQRGDD**pT**D**pS**DNEAVNAQAR	-5.09	-3.61
LinJ.25.1120	hypothetical protein, conserved	**pS**NFG**pS**RVDLHNLGDLSR	-5.67	-2.07
LinJ.25.1120	hypothetical protein, conserved	SNFG**pS**RVDLHNLGDLSR	-5.67	-4.08
LinJ.32.0370	hypothetical protein, conserved	RPAGLSPGAAAALAADEKQA**pS**CAACH	-5.76	-6.45
LinJ.15.0060	hypothetical protein, conserved	KPL**pT**PPRLILAPPTALGQ**pS**PDVKPK	-5.95	-6.25
LinJ.13.0700	hypothetical protein, conserved	R**pS**RSPVAWTASIGGAAK	-6.51	-3.13
LinJ.13.0620	ubiquitin-like protein	RSDDSLQFEQDASQQPT**pS**PLLPPQAPVKAPAASAFDR	-6.7	-2.37
LinJ.35.0300	hypothetical protein, conserved	AP**pS**PDFLGSQLLAEYRGEAASLQLAK	-6.86	-5.37
LinJ.18.0590	RNA binding protein, putative	A**pS**PQQQAAVQSAPGKIEYLR	-6.94	-4.48

LinJ.23.1190	hypothetical protein, unknown function	NE**pS**DDMERQADEKR	-7.31	-7.1

Di methylation of tryptic peptides extracted from *L. donovani* promastigotes 15 min after arginine deprivation initiated. Phosphopeptide enrichment, mass spectrometry and data analysis is described in detail in Experimental Procedures
